# Distribution and associations of intraocular pressure in 7- and 12-year-old Chinese children: The Anyang Childhood Eye Study

**DOI:** 10.1371/journal.pone.0181922

**Published:** 2017-08-17

**Authors:** Shuning Li, Shi-Ming Li, Xiao-lei Wang, Meng-Tian Kang, Luo-Ru Liu, He Li, Shi-Fei Wei, An-Ran Ran, Siyan Zhan, Ravi Thomas, Ningli Wang, the Anyang Childhood Eye Study Group

**Affiliations:** 1 Beijing Tongren Eye Center, Beijing Tongren Hospital, Beijing Ophthalmology & Visual Science Key Lab, Beijing Institute of Ophthalmology, Capital Medical University, Beijing, China; 2 Department of Ophthalmology, Beijing Friendship Hospital, Capital Medical University, Beijing, China; 3 Anyang Eye Hospital, Anyang, Henan, China; 4 Department of Epidemiology and Health Statistics, Peking University School of Public Health, Beijing, China; 5 Queensland Eye Institute, Brisbane, Australia; 6 University of Queensland, Brisbane, Australia; Soochow University Medical College, CHINA

## Abstract

**Purpose:**

To report the intraocular pressure (IOP) and its association with myopia and other factors in 7 and 12-year-old Chinese children.

**Methods:**

All children participating in the Anyang Childhood Eye Study underwent non-contact tonometry as well as measurement of central corneal thickness (CCT), axial length, cycloplegic auto-refraction, blood pressure, height and weight. A questionnaire was used to collect other relevant information. Univariable and multivariable analysis were performed to determine the associations of IOP.

**Results:**

A total of 2760 7-year-old children (95.4%) and 2198 12-year-old children (97.0%) were included. The mean IOP was 13.5±3.1 mmHg in the younger cohort and 15.8±3.5 mmHg in older children (P<0.0001). On multivariable analysis, higher IOP in the younger cohort was associated with female gender (standardized regression coefficient [SRC], 0.11, P<0.0001), increasing central corneal thickness (SRC, 0.39, P<0.0001), myopia (SRC, 0.05, P = 0.03), deep anterior chamber (SRC, 0.07, P<0.01), smaller waist (SRC, 0.07, P<0.01) and increasing mean arterial pressure (SRC, 0.13, P<0.0001). In the older cohort, higher IOP was again associated with female gender (SRC, 0.16, P<0.0001), increasing central corneal thickness (SRC, 0.43, P<0.0001), deep anterior chamber (SRC, 0.09, P<0.01), higher body mass index (SRC, 0.07, P = 0.04) and with increasing mean arterial pressure (SRC, 0.09, P = 0.01), age at which reading commenced (SRC, 0.10, P<0.01) and birth method (SRC, 0.09, P = 0.01), but not with myopia (SRC, 0.09, P = 0.20).

**Conclusion:**

In Chinese children, higher IOP was associated with female gender, older age, thicker central cornea, deeper anterior chamber and higher mean arterial pressure. Higher body mass index, younger age at commencement of reading and being born of a caesarean section was also associated with higher IOP in adolescence.

## Introduction

Intraocular pressure (IOP) is an important characteristic of eye growth.[1] Compared to adults, there is a relative paucity of information, especially population based data on the association of intraocular pressure (IOP) with myopia and other factors in children.REFs IOP has been reported to be associated with factors such as central corneal thickness (CCT), refractive error as well as systemic factors including age, sex, ethnicity, blood pressure and prematurity [2–11]. However, other reports have not found any associations between IOP and refractive error [12–15], age [9,13,15,16], ethnicity [17], sex [5,9,13,15,18,19] or even CCT [17]. These conflicting results may be due to measurement methods, sample sizes, age ranges, ethnic diversity as well as interactions between these and ocular factors [2,9,20].

Myopic eyes have been reported to have a higher IOP than non myopic eyes and it has been hypothesized that a higher IOP may affect the development of myopia in children [4,21,22]. Other reports have not supported an association between IOP and refractive error/axial length [12,13,23] or change in these parameters on follow-up [5].

The purpose of this study is to report the IOP and its associations, including myopia in a large population based sample of Chinese children.

## Materials and methods

The Anyang Childhood Eye Study (ACES) is a school-based cohort study that aims to report the prevalence, incidence and progression of myopia in two cohorts (grade 1 and grade 7) Chinese students in the urban areas of Anyang, Henan Province, Central China. Details of the methodology have been reported previously [24–26]. The grade.

7 students were recruited from October 2011 to December 2011. The ACES adhered to the Declaration of Helsinki and was approved by the ethics committee of Beijing Tongren Hospital, Capital Medical University. Each student was asked for verbal assent and informed written consent was obtained from at least one parent. The authors had access to information that could identify individual participants during or after data collection.

IOP was recorded with a non-contact tonometer (Havitz, HNT-7000) in both eyes. Three measurements were obtained for each eye, and the mean value was recorded. CCT measurements were obtained with the iVue 100 (Optovue Inc, Fremont, California, USA) [27–29]. The measurements were made as recommended by the manufacturer: the children fixated on an internal light in the machine for the few seconds required to generate automatic measurements [28]. Ocular biometric parameters were measured using IOL Master (Carl Zeiss, Meditec AG Jena, Germany); the average of five measurements was averaged.

Cycloplegic autorefraction was performed using an autorefractor (HRK-7000A, HUVITZ, South Korea). Cycloplegia was achieved by instilling 2 drops of 1% cyclopentolate (Alcon) and 1 drop of Mydrin P (Santen, Japan) at 5-minute intervals preceded by a single drop of topical anesthetic agent (Alcaine, Alcon). A third drop of cyclopentolate was administrated if the pupillary light reflex was still present and / or the pupil size was < 6.0 mm. Three auto-refraction readings were taken and the average was recorded. Emmetropia, myopia and hyperopia were defined as the cycloplegic spherical equivalent (SE) of +1.0 D to -0.5 D, < -0.5D and ≥+1.0D, respectively.

Data on number of myopic parents (0, 1 2), birth length, birth weight, birth method (normal or caesarean), gestational week, smoking during pregnancy (yes or no), living with smoker during pregnancy (yes or no), doing Chinese eye exercise (yes or no), age at commencement of reading, maximum time spent on continuous reading at one session, distance between face and book when reading and writing, headache during near work (yes or no), books read per week, time spent on near work and outdoors, lights on while sleeping at night (yes or no), parents smoking status (yes or no), water intake per day, frequency for drinking tea or coffee, favorite beverage, and age of myopia onset were obtained using questionnaires that have been published elsewhere [24].

### Statistical analysis

As the IOP of right and left eyes were highly correlated (Pearson’s correlation coefficient *r* = 0.8), only the data from right eyes were used for analysis. Refractive error was analyzed as spherical equivalent (SE), calculated as sphere plus half cylinder. Statistical analysis was performed using Statistical Analysis System Software (version 9.1.3, SAS Institute, Inc., Cary, NC, USA) and values were reported as mean ± standard deviation. Independent t-test was performed to evaluate the differences in baseline characteristics of the two groups of children. Univariable regression analysis was performed to assess the associations between IOP and ocular and general parameters. This was followed by multivariable regression analysis using the variables that had a P value less than 0.1 in univariable analysis or had any factor with biological plausibility. P≤0.05 was considered to be statistically significant.

## Results

Data was available for 2760 of the 2893 grade 1 students (95.4%) and 2198 of the 2267 grade 7 students (97.0%) examined (Table 1) [24]. Some students or their parents refused to undergo the measurement of IOP. The mean age of grade 1 and 7 children was 7.1 and 12.7 years, respectively. The older cohort had a higher IOP (15.8 mm Hg vs. 13.5 mm Hg, P<0.01), longer axial length (24.1 mm vs. 22.7 mm, P<0.01), higher myopia (-1.5 D vs. +0.9 D, P<0.01), deeper anterior chambers (3.6 mm vs. 2.9 mm, P<0.01), higher proportions of non-myopic parents, higher blood pressure and higher anthropometric parameters than the 7-year-olds. In both cohorts the IOP was marginally higher in girls than in boys and in myopes compared to emmetropes or hyperopes.

**Table 1:** Baseline characteristics of grade 1 (n = 2760) and grade 7 (n = 2198) children.

	Young group	Older group	*P*
Age (y)	7.1±0.4	12.7±0.5	<0.01
Gender (female, %)	1154(41.8)	1072(48.8)	<0.01
SE (D)	0.9±0.9	-1.5±2.0	<0.01
Axial length (mm)	22.7±0.7	24.1±1.0	<0.01
Anterior Chamber depth (mm)	2.9±0.2	3.6±0.3	<0.01
Intraocular pressure (mmHg)	13.5±3.1	15.8±3.5	<0.01
Boys	13.3±3.0	15.5±3.4	
Girls	13.8±3.2	16.2±3.5	
Hyperopia	13.3±3.0	14.3±3.3	
Emmetropia	13.7±3.1	15.3±3.3	
Myopia	14.0±3.1	16.2±3.5	
Central corneal thickness (um)	540±31	538±34	0.07
Parental myopia (n, (%))			<0.01
None	1307 (60.3)	1282 (69.7)	
Either	666 (30.7)	440 (23.9)	
Both	194 (9.0)	117 (6.4)	
Systolic blood pressure (mmHg)	97.3±10.2	105.5±11.6	<0.01
Diastolic blood pressure (mmHg)	58.3±9.4	66.1±9.8	<0.01
Mean arterial pressure (mmHg)	71.3±9.0	79.2±9.4	<0.01
Height (m)	123.5±5.5	155.0±7.3	<0.01
Weight (kg)	24.6±4.8	47.7±10.9	<0.01
BMI (kg/m2)	16.1±2.4	19.8±3.7	<0.01

The results of univariable regression analysis in the two groups are shown in Table 2. In the younger group of children, IOP was significantly associated with gender, number of myopic parents, systolic pressure, diastolic pressure, mean arterial pressure, time outdoors, CCT, spherical equivalent, axial length, anterior chamber depth. In the older group IOP was also associated with age, gender number of myopic parents, birth method, systolic pressure, diastolic pressure, mean arterial pressure, CCT, spherical equivalent, axial length and anterior chamber depth. It was also associated with the age at which they started reading and hours spent sleeping.

**Table 2:** Associations of IOP on univariable regression.

Parameters	P value	
	Grade 1 Children	Grade 7 Children
**General Parameters**		
Age	0.21	0.05
Gender#	< .0001	< .0001
Number of myopic parents†	0.0089	0.002
Height	0.27	0.01
Weight	0.99	< .0001
Waist	0.047	0.06
BMI	0.57	0.0004
Birth length	0.09	0.44
Birth weight	0.10	0.57
Birth method**	0.047	0.0003
Gestational weeks	0.02	0.41
Smoking during pregnancy*	0.85	0.67
Living with smoker during pregnancy*	0.27	0.98
Doing Chinese eye exercise*	0.56	0.82
Systolic pressure	< .0001	< .0001
Diastolic pressure	< .0001	0.0009
Mean arterial pressure	< .0001	< .0001
Age at commencement of reading	0.99	0.003
Maximum time spent reading continuously	0.26	0.13
Distance between face and book when reading /writing	0.37	0.17
Headache during near work*	0.53	0.47
Books read per week	0.11	0.62
Time spent on near work	0.09	0.43
Time spent outdoors	0.03	0.80
Time spent sleeping	0.0497	0.02
Lights on while sleeping at night*	0.75	0.84
Parents smoking status*	0.17	0.008
Water intake per day	0.60	0.04
Freqency for drinking tea	0.74	0.59
Favorite beverage	0.94	0.17
Frequency for drinking coffee	0.25	0.87
**Ocular Parameters**		
Central corneal thickness	< .0001	< .0001
Spherical equivalent	< .0001	< .0001
Axial length	0.03	< .0001
Anterior chamber depth	0.01	0.0001
Dominant eye	0.08	0.03
Age of myopia onset	0.42	0.09

# male as reference

† 0 as reference

**Normal labour as reference

* Answer ‘no’ as reference

The results of multivariable regression analysis are shown in Table 3. In grade 1 children a higher IOP was associated with female gender, thicker CCT, deeper anterior chamber, higher mean arterial pressure, higher myopia and smaller waist size. In the grade 7 children it was also associated with female gender, thicker CCT, deeper anterior chamber, higher mean arterial pressure but also with higher body mass index, younger age at onset of reading and birth method of caesarean (normal labour as reference).

**Table 3:** Associations of IOP on multivariable regression analysis.

Parameters		Grade 1 Children			Grade 7 Children	
	Parameter estimate	Standardized regression coefficient	P value	Parameter estimate	Standardized regression coefficient	P value
Gender	0.68828	0.11038	< .0001	1.15001	0.16014	< .0001
Central corneal thickness	0.03934	0.39056	< .0001	0.04598	0.42841	< .0001
Anterior chamber depth	0.87725	0.06798	0.0055	1.32966	0.09160	0.0078
Mean arterial pressure	0.04460	0.13181	< .0001	0.03472	0.08854	0.0107
Spherical equivalent	-0.17213	-0.05227	0.0276			
Waist	-0.03562	-0.07103	0.0018			
Body mass index (BMI)				0.07044	0.07207	0.0406
Age at commencement of reading				-0.19314	-0.09629	0.0043
Birth method*				0.70524	0.08643	0.0100

* Normal labour as reference

## Discussion

In this large sample of Chinese children, we found that the grade 7 children had a higher IOP (2.3 mmHg) compared to those in grade 1. In both groups, a higher IOP was associated with being girls, thicker central cornea, deeper anterior chamber and higher blood pressure. In young children, higher IOP was also associated with more myopia and smaller waist. In older children, higher IOP was also associated with younger age of independent reading and caesarean.

The IOP in children of different ethnicities (and adult Chinese) are shown in Table 4. The mean IOP in our study was similar to that of children from Japan[30], Czechoslovakia [31], Malaysia [32] and Singapore (including Chinese, Malays and Indians) [13,33], but was lower than that reported in Turks (16.7 and 17.5 mm Hg) [34,35], USA (black children, 19.3 mm Hg; White children, 17.7 mm Hg) [16], UK (16.7 mm Hg) [36], as well as children from Eastern China (17.6 mm Hg) [37]. While some of these differences could be related to the measurement methods, structural differences between different racial groups also likely play a role. Interestingly the mean IOP in our grade 7 children was similar to that of adult Chinese reported from Northern China [38,39], Southern China [40,41], and Singapore [42]. The measurement technique was not uniform, but the data suggest that adult levels of IOP could be achieved by about 12 years of age. If confirmed baseline measurements at a younger age may be useful to identify true change in IOP unrelated to age.

**Table 4:** Mean intraocular pressure (IOP) obtained in children of different ethnicities and adult Chinese.

Study	Ethnicity	Age (years)	Method	Mean±SD
Li et al. (current study)	Northern Chinese	7.1±0.4	Non-contact tonometry	13.5±3.1
		12.7±0.5	Non-contact tonometry	15.8±3.5
Jiang et al.[37]	Eastern Chinese	4–18	Non-contact tonometry	17.6±2.7
Hikoya et al.^[^^30^^]^	Japanese	8 months–18	Tono-Pen	13.9±2.4
Muir et al.^[^^44^^]^	Black	5–17	Goldmann applanation and Tono-Pen	19.3±6.0
	White			17.7±4.2
Yildirim et al.^[^^35^^]^	Turkish	10.1±1.6	Noncontact tonometry	16.7±2
Sahin et al.^[^^34^^]^	Turkish	7–12	Tono-Pen	17.47±2.7
Osmera et al.^[^^31^^]^	Czech	7–17	Goldman applanation Tonometry	14.5±2.6
Lee et al.^[^^13^^]^	Singaporean Chinese	9–11	Non-contact tonometry	16.6±2.7
Doughty et al.^[^^36^^]^	White	5–15	Non-contact tonometry	16.7±2.9
Lim et al.^[^^33^^]^	Singaporean (Chinese, Malays and Indians)	13.97±0.9	Ocular Response Analyzer	15.12±2.84
Heidary et al.^[^^32^^]^	Malay	12.27±2.76	Non-contact tonometry	15.65±3.05
Zhao et al.^[^^38^^]^	Northern Chinese	50+	Perkins	13.5±2.2
Xu et al.^[^^45^^]^	Northern Chinese	40+	Non-contact tonometry	16.1±3.4
He et al.^[^^40^^]^	Southern Chinese	50+	Tonopen	15.2±3.1
Lin et al.^[^^41^^]^	Southern Chinese	65+	Non-contact tonometry	12.9±3.1
Foster et al.^[^^42^^]^	Singaporean Chinese	40+	Goldman applanation Tonometry	15.3

The COMET study reported a slightly higher IOP (0.66 mm Hg) in boys compared to girls [5]. Three years later the findings were reversed, with IOP lower by 0.57 mm Hg in boys [2]. We report lower IOP in boys compared to girls, but the differences were small, 0.5 mmHg in the 7-year-olds and 0.7 mmHg in the 12-year-olds. While gender was identified as a factor associated with IOP on multivariable regression, the differences are small and probably insignificant.

The CCT was similar in both groups and showed a positive association with IOP (an increase of 4~5 mm Hg in IOP for every 100 μm in CCT), consistent with most previous reports [9,16,43]. Central corneal thickness has been previously reported to be greater in children with ocular hypertension than in controls.

In our study, IOP was higher in myopic eyes than in emmetropic or hyperopic eyes. While the absolute difference was small in grade 1 children, this increased to 1 mmHg in grade 2 children and may suggest a trend. A significant negative association between IOP and spherical equivalent was found in univariable analysis in both groups of children. On multivariable regression this association persisted only in the younger children. IOP was positively associated with deeper anterior chamber in both groups of children (about 1 mm Hg/mm).

We also found that children with who started reading earlier in life had a higher IOP. This supports other reports that time spent indoors reading or writing was associated with a higher IOP [37]. The same study also reported an association between higher IOP and longer axial length [37], which is similar to our findings of deeper anterior chamber and higher IOP. These results confirm that a higher IOP is associated with higher myopia and relevant ocular parameters but its role as a causative factor remains unclear.

Higher IOP was associated with higher mean arterial pressure in both young and older children: a 10 mm Hg increase in mean arterial pressure was associated with about a 0.4 mm Hg increase in IOP. This is similar to what has been reported in adult Chinese, Foster et al [42]. found that a 10 mm Hg increase in blood pressure was associated with 0.3 mm Hg in IOP.

Birth by caesarean section was found to be associated with higher IOP compared with normal labour. While this could be chance finding, it is possible that children delivered by caesarean section are more likely to be premature and myopic, and have higher IOP via that association. We do accept that some factors may be significant by chance alone.

The strengths of the present study are the population based large sample size, two groups of children with different mean age and multiple well documented factors included for analysis and the high rate of participation. There are several limitations too. Firstly, a non-contact tonometer rather than the Goldmann tonometer was used to measure IOP. The non-contact tonometer was selected because it is quick, easy to use on children with a lower risk of disease transmission and has been used in other studies in children. Next, the two groups covered two different age groups with no children in between; this limits extrapolation of the effect of age on IOP. Also, as it involved a different cohort, the effect of age is actually counterfactual: ideally we should follow the same cohort to detect this change.

In summary, in grade 1 and grade 7 Chinese children, IOP was associated with gender, age, central corneal thickness, anterior chamber depth and blood pressure. Follow up of these two cohorts to further assess these associations and the role of IOP in the development of myopia is planned.

## Supporting information

S1 Table The underlying participant-level data. (XLS)
